# Food price volatility

**DOI:** 10.1098/rstb.2010.0139

**Published:** 2010-09-27

**Authors:** C. L. Gilbert, C. W. Morgan

**Affiliations:** 1 Department of Economics, University of Trento, Trento, Italy; 2 Department of Economics, University of Nottingham, Nottingham, UK

**Keywords:** food, volatility, rice

## Abstract

The high food prices experienced over recent years have led to the widespread view that food price volatility has increased. However, volatility has generally been lower over the two most recent decades than previously. Variability over the most recent period has been high but, with the important exception of rice, not out of line with historical experience. There is weak evidence that grains price volatility more generally may be increasing but it is too early to say.

## 1. Introduction

**Figure 1.**
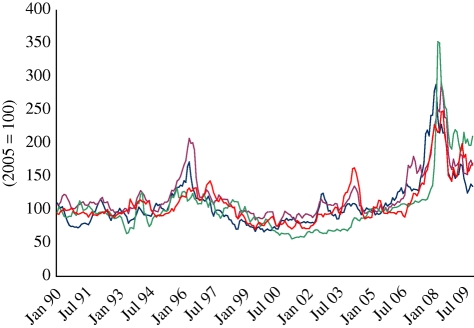
Grains price index numbers (2005 = 100), 1990–2009. Dark grey line, wheat; brown line, maize; green line, rice; red line, soya beans.

World dollar prices of major agricultural food commodities (‘food prices’ in what follows) rose dramatically from late 2006 through to mid-2008. Prices collapsed dramatically in the second half of 2008 with the onset of the financial crisis. This episode is often referred to as the ‘2008 price spike’. Prices partially recovered in the second half of 2009 to levels that generally exceed pre-spike values. Figure 1 shows (nominal) monthly prices for major grains and oilseeds over the period 1990–2009.

A number of authors have discussed the factors that lie behind the 2008 price spike (Abbot *et al*. 2008; Mitchell 2008; Cooke & Robles 2009; Gilbert 2010*a*). A large number of potential explanations are available. Those given greatest prominence are:

— rapid economic growth, particularly in China and other Asian economies, emphasized by Gilbert (2010*a*);
— decades of underinvestment in agriculture (World Bank 2007);
— low inventory levels;
— poor harvests, in particular in Australia;
— depreciation of the US dollar, emphasized by Abbot *et al*. (2008);
— diversion of food crops into the production of biofuels, emphasized by Abbot *et al*. (2008) and Mitchell (2008); and
— speculative influences, emphasized by Cooke & Robles (2009) and Gilbert (2010*a*,*b*).

We do not join this debate. Instead, we ask whether food prices have become more variable. Was the 2008 price spike a ‘one-off’ event without implications for the longer term, or does it signal the initiation of a more volatile period in which price spikes of this sort will become more frequent occurrences? Previous periods of high volatility have prompted the same questions but the historical experience has generally been that periods of high volatility have been relatively short and interspaced with longer periods of market tranquillity. It would therefore be wrong simply to extrapolate recent and current high volatility levels into the future. However, it remains valid to ask whether part of the volatility rise may be permanent.

The structure of the paper is as follows. Section 2 contains a historical review of food price volatility. Section 3 looks at volatility determinants. Section 4 then considers the likely future evolution of food price volatility while §5 considers the effects of heightened volatility. Rice is discussed in §6 on the basis that it differs in significant ways from other food commodities. Section 7 considers public policy with the objective of moderating volatility or offsetting its effects, and §8 concludes.

## 2. Historical review

Volatility is a directionless measure of the extent of the variability of a price or quantity. It follows that volatility measures derive from the second moment of the distribution of the price or quantity in question, or transformations thereof. Economists generally focus on the standard deviation of logarithmic prices since this is a unit-free measure. For low levels of volatility, the log standard deviation is approximately equal to the coefficient of variation.

Economic series typically exhibit trends. Measurement of volatility therefore requires the series to be detrended since otherwise trend movements will be included in the volatility measures. Because trends are rarely linear and deterministic (Kim *et al*. 2003; Kellard & Wohar 2006), detrending requires a trend model that implies a judgemental trade-off between attribution of variability to the trend itself and to variation about the trend. The volatility measure can therefore depend on the choice of the trend model in an undesirable manner. In looking at price volatility, economists often circumvent these issues by measuring volatility as the standard deviation of price returns, i.e. the standard deviation of changes in logarithmic prices. We adopt this standard measurement convention.

Academic and policy analyses have tended to focus on price levels rather than volatilities. An exception is Gilbert (2006) who showed that agricultural price volatility was low in the 1960s but was higher in the 1970s and the first half of the 1980s. Volatility fell back in the second half of the 1980s and the 1990s but remained well above its 1960s level.

**Figure 2.**
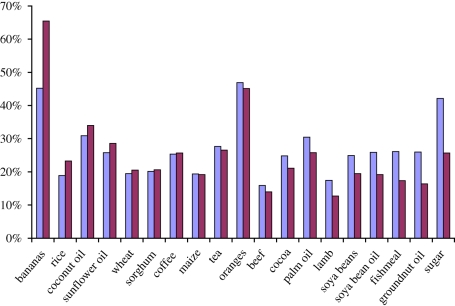
Changes in volatility over time. Lavender coloured bars, 1970–1989; magenta bars, 1990–2009.

Table 1 updates table 4 of Gilbert (2006) looking from 1970 to 2009. The sample is divided at the end of 1989, which is the half-way point in the sample. The first column of the table reports the volatility estimate for the commodity over the entire 40 year period. The second column gives the estimates for 1970–1989 and 1990–2009. The third column reports the standard *F*-test for variance equality. The test outcome is summarized in the final column. Figure 2 shows the same figures graphically, with the commodities ordered by the extent to which volatility increased between the two periods.

**Table 1.** Price volatilities 1970–2009. Standard deviations of logarithmic changes in monthly average real US dollar prices at an annual rate, January 1970–December 2009. Nominal prices are deflated by the US PPI (all items). *p*-values are given in parentheses. Sources: IMF, *International Financial Statistics*, except coffee (International Coffee Organization).

	1970–2009	1970–1989	1990–2009	equality test	(*p*-value)	
beverages plus sugar (%)						
cocoa	23.1	24.8	21.1	1.38	(0.7)	significant fall
coffee	25.5	25.4	25.7	1.03	(42.6)	insignificant rise
sugar	35.0	42.2	25.7	2.69	(<0.1)	significant fall
tea	27.1	27.6	26.5	1.08	(26.6)	insignificant fall
grains (%)						
maize (corn)	19.3	19.4	19.2	1.02	(44.2)	insignificant fall
rice	21.1	18.9	23.3	1.52	(0.1)	significant rise
sorghum	20.4	20.2	20.6	1.05	(36.1)	insignificant rise
soya beans	22.4	24.9	19.5	1.64	(<0.1)	significant fall
wheat	20.0	19.5	20.5	1.11	(21.2)	insignificant rise
fats and oils (%)						
coconut oil	32.4	30.9	34.0	1.21	(7.0)	insignificant rise
groundnut oil	21.8	26.0	16.4	2.52	(<0.1)	significant fall
palm oil	28.2	30.4	25.8	1.40	(0.5)	significant fall
soya bean oil	22.8	25.9	19.2	1.83	(<0.1)	significant fall
sunflower oil	27.2	25.8	28.6	1.23	(5.8)	insignificant rise
meats and fish (%)						
beef	15.0	15.9	14.0	1.29	(2.4)	significant fall
lamb	15.3	17.4	12.7	1.88	(<0.1)	significant fall
fishmeal	22.2	26.1	17.3	2.27	(<0.1)	significant fall
fresh fruit (%)						
bananas	56.1	45.2	65.5	2.10	(<0.1)	significant rise
oranges	46.0	46.9	45.1	1.08	(27.6)	insignificant fall

From the first column of table 1, we see that agricultural volatilities have been lowest for grains and meats and highest for fresh fruit. Fruit is perishable and storage, which can limit volatility, plays a more limited role for fruits than for the other commodities considered in the table—see the discussion in §3. Columns 2–4 of table 1 show that there was a statistically significant rise in volatility for only two commodities—bananas and rice. By contrast, nine commodities saw statistically significant falls in volatility—cocoa, soya beans, sugar, three vegetable oils (soya bean, groundnut and palm) and the three meat and fish products (beef, lamb and fishmeal). Overall, therefore, the most recent two decades have seen lower levels of agricultural volatility than in those of the 1970s and 1980s, with rice constituting the main exception to this tendency. These findings are in line with those of Balcombe (2009) who also failed to find evidence of any general increase in volatilities.

In splitting the sample at the end of the 1980s, the tests reported in table 1 provide a relatively crude indication of whether volatilities have been changing. It is arguable that it is the high volatility levels of the most recent years that are out of line with past experience. This is difficult to judge because volatility itself is highly variable over time. Furthermore, periods of high volatility tend to bunch. One way of posing the question in relation to recent levels of volatility is to estimate a volatility model.

The generalized autoregressive conditional heteroscedasticity (GARCH) model is now the standard procedure for modelling volatility in financial markets (Engle 1982; Bollerslev 1986). GARCH specifies an autoregressive moving average process for the variance (scedastic) process followed by a time series to yield an estimate of the conditional variance of the process at each date in the sample. In Gilbert & Morgan (2010), we use the first-order GARCH(1,1) framework to ask whether there was an upward shift in the mean of the scedastic process over the period 2007–2009. The question may be paraphrased as asking whether the conditional volatility of food prices was higher from 2006 or whether we simply observed a number of high prices, leaving expected volatility unchanged.

Results are summarized in table 2. They vary across commodity groups. At the 5 per cent level, the mean of the conditional volatility process only increased significantly for soya bean oil. (Bananas show a significant volatility decrease.) However, the estimated increase in conditional volatility is positive for all five grains and all five vegetable oils, and the increases are significant at the 10 per cent level for soya beans and groundnut oil as well as for soya bean oil. There is no systematic pattern in the change in conditional volatility for the remaining nine food commodities.

**Table 2.** GARCH dummy variable coefficients. The table reports the estimated *t*-statistic on a dummy variable with value 1 from January 2007 included in a GARCH(1,1) model for the price series over the period January 1990–December 2009 (240 observations). The first-order (mean) process is MA(1). Statistically significant *t-*statistics (at the 5% level and a one-sided alternative) are shown in italic. See Gilbert & Morgan (2010) for further details.

cocoa	0.85	maize (corn)	0.44	coconut oil	0.18	bananas	*−2.07*
coffee	−1.17	rice	0.91	groundnut oil	1.94	oranges	−0.69
sugar	0.88	sorghum	0.28	palm oil	1.46	beef	0.53
tea	0.90	soya beans	1.79	soya bean oil	*2.16*	lamb	−1.36
		wheat	1.66	sunflower oil	1.21	fishmeal	−1.08

To summarize, this analysis has generated two conclusions:

— Agricultural price volatility was generally lower over the past two decades than in the 1970s and 1980s, the major exception being rice.
— Although the prices of many food products exhibited high variability over the 3 year periods 2007–2009, conditional variances increased significantly for groundnut oil, soya beans and soya bean oil.

It follows that, although there has not been any general tendency for food price volatility to increase over the most recent years, volatilities of the most important grains have increased. While this does not imply that these volatilities will remain high, it does underline concern that there is an increased likelihood of further sharp price movements for these products.

## 3. The causes of food price volatility

Agricultural prices vary because production and consumption are variable. Economists distinguish between predictable and unpredictable variability, the latter being characterized in terms of shocks. Shocks to production and consumption transmit into price variability. Production can vary either because of variations in area planted or because of yield variations, typically owing to weather. Consumption varies because of changes in incomes, changes in prices of substitutes and shifts in tastes. It is generally supposed that the most important source of price variability in agriculture is weather shocks to agricultural yields. Nevertheless, demand shocks, in particular income shocks (Gilbert 2010*a*) and policy shocks (Christiaensen 2009), may also play an important role.

The extent to which given production and consumption shocks translate into price volatility depends on supply and demand elasticities, which, in turn, reflect the responsiveness of producers and consumers to changes in prices. It is generally agreed that these elasticities are low over the short term, in particular within the crop year. Farmers cannot harvest what they have not planted and will almost invariably harvest everything that they have planted. Consumers are reluctant to revise habitual dietary patterns and, in poor countries, they may have few alternatives. Furthermore, the commodity raw material may comprise only a small component of many processed foods, with the consequence that even large commodity price rises have a small impact on final product prices.

Stockholding causes volatility to bunch. When stocks are low, relatively small production or consumption shocks can have large price impacts but when they are high, the reverse is the case. Moreover, once stock levels become high, they will remain high until consumption has exceeded production for sufficient time to absorb past surpluses. Stockholding therefore results in a cyclical pattern in prices and volatilities even if supply and demand shocks are independent over time. World grain stocks fell to low levels by 2006, and this is seen as one cause of recent high grains price volatility. Since it takes time to rebuild stocks, it is possible that volatility levels will remain high over the next few years. But this does not imply that volatilities will be permanently higher.

Other factors may also be important in either amplifying or attenuating volatility. Stockholding will reduce volatility so long as stocks are accumulated in periods of excess supply and released in times of excess demand. However, stockholding is more effective in reducing the extent of price falls in the event of positive supply shocks (abundant harvests) than in reducing the extent of price rises in the event of shortfalls since destocking depends on the existence of a carryover from previous years. Stockholding therefore reduces volatility but also gives a positive skew to the price distribution (Wright & Williams 1991; Deaton & Laroque 1992).

Speculation is a second factor that may have either a positive or a negative impact on volatility. Speculation may be either through stockholding or through purchase and sale of commodity futures or other derivative contracts. However, not all futures markets transactions are speculative—the standard regulatory distinction between hedging, in which supply chain agents attempt to offset risk exposure through futures transactions, and speculation is that speculators are ‘non-commercials’, i.e. they do not have any involvement in the physical commodity trade. Commodity futures markets are seen as providing a structure in which risk is transferred from commercial to non-commercial traders, i.e. from hedgers to speculators. In assuming this price risk, speculators provide the market liquidity that enables hedgers to find counterparties in a relatively costless manner.

By analogy with insurance markets, in aggregate, speculators will expect to profit and hedgers to pay for this risk transfer. The traditional view among economists is that speculation will tend to be stabilizing (i.e. volatility reducing) because destabilizing speculation will be unprofitable and will therefore not persist (Friedman 1953). However, much speculation is undertaken by trend-following commodity trade advisors or amateur traders, and there is a worry that their extrapolatively based actions may result in self-fulfilling beliefs—if identified as a nascent trend, a randomly induced price rise will generate further buying, thereby reinforcing the initial movement (De Long *et al*. 1990; Irwin & Yoshimaru 1999; Irwin & Holt 2004; Gilbert 2010*b*).

More recently, a significant group of institutional investors have started to invest in commodity futures through index-based swap transactions as a portfolio diversification strategy and to assume exposure to the commodity ‘asset class’. In agricultural futures markets, these positions are often large in relation to total activity—up to 40 per cent of market open interest (Gilbert 2010*b*). Differently from traditional speculation, these positions are relatively long term and are predominantly long, i.e. they involve purchase of futures contracts, which are then held as long-term investments. The sharp rise in index-based investment in commodity futures over the past five years may therefore be seen as a positive shock to inventory demand. Gilbert (2010*a*) argues that this shock was a significant contributory factor to the 2007–2008 food price spike; see also US Senate Permanent Subcommittee on Investigations (2009).

Food price volatility arises from shocks that can come from a number of sources, with the impact being felt differently in each separate commodity market. On some occasions, these shocks will be correlated. Often, this will be the case if common factors simultaneously affect a range of different markets, perhaps including non-agricultural markets. This appears to have been the case in 2007–2008 when most agricultural prices and many non-agricultural prices (energy, metals and freight rates) rose simultaneously. It was also the case in the 1973–1974 food price spike. In such cases, it appears likely that there are common causal factors. There is less agreement in the identity of these causal factors but demand growth, high oil prices perhaps generating demand for grains as biofuel feedstocks, dollar depreciation and futures market speculation are all candidates in this regard (Cooper & Lawrence 1975; Baffes 2007; Abbot *et al*. 2008; Mitchell 2008; Gilbert 2010*b*).

## 4. Consequences of food price volatility

Our focus in this discussion is grains and, to a lesser extent, vegetable oils since these are overall the most important food crops. Grains are the major staple food across the globe and also are an input into the production of meat products. As such, they are key within the food price volatility question. Within the grains group, we can distinguish between:

— wheat, which is the most important grain in temperate regions;
— maize, which is grown in both temperate and tropical zones; white maize is the major staple food in eastern and southern Africa while yellow maize is the major biofuel feedstock in the United States and, increasingly, China;
— rice, which is the staple food through most of Asia and is also important in Africa and Latin America; and
— soya beans, which are important both as an animal feedstock and, when crushed, as a vegetable oil.

Direct consumption of grains declines as societies become richer. The consequence is that the impact of high and volatile grain prices concentrates on the poorer rather than the richer economies and on the poor rather than the rich within each economy. In general terms, it is probably correct to argue that, in the richer developed economies such as Britain, energy price volatility is more problematic than food price volatility. Food price volatility therefore has a greater impact on the developing world, where, depending on the region, maize and rice are the most important food staples. Meat consumption, by contrast, rises as consumers become richer, at least at low and moderate income levels, and this translates into a greater indirect dependence on grains as animal feedstocks. The indirect impact of grains price volatility through meat prices is therefore most acute at middle levels of income.

The impact of food price volatility can be viewed at both the economy level and at the individual (producer and consumer) level, although the impact will depend on which economy and which individuals are being examined. Focusing on the economy level first, there are a number of key factors that will affect the way food price volatility will create an impact. Virtually all economies trade in food—as importers and/or exporters—and thus volatility in world food prices will potentially have trade bill effects, the net outcome of which will depend on the country's net food export position and the extent to which it is integrated in world markets. As such, it is unsurprising to note that a country-by-country approach to evaluating the effect of food price volatility would need to be carried out before precise impacts could be measured and even then, specific periods of time would have to be identified over which the effects were to be measured. However, it is possible to review some of the generic outcomes alongside case studies of particular countries.

Importing, richer nations are concerned about food price volatility in terms of the impact it might have on consumer price inflation and, to a lesser extent, by balance of trade effects. As world commodity prices generally rise, food prices included, domestic price levels could rise with fears of price–wage spirals being set off (Bloch *et al*. 2007). Mundlak & Larsen (1992) explore the transmission of world prices to domestic levels and the null hypothesis of the law of one price rarely holds owing to many factors, not least of which are impact of exchange rates and degrees of imperfect competition within domestic supply chains. It is possible to characterize richer nations as being more open to world price effects given established trading policies, which could suggest a greater concern over volatility, but this is dampened by the relatively low expenditure on food as a proportion of national income. The same concerns arise with respect to oil price volatility but pass-through has been low over the most recent decade.

Looking at individuals in richer nations, consumers of food, now largely in the form of processed food products, are affected to the extent that world agricultural prices are transmitted into the prices paid for products in retail outlets. Retail sectors are often imperfectly competitive (Clarke *et al*. 2002) and thus pass-through is often incomplete, dampening volatility effects. More pertinent is the possible link to rising wage demands to compensate for higher food prices, but this is now a relatively weak link given the relatively low proportion of household income spent on food (10–15% in many countries is typical). Perhaps of some interest is the relative impact on poorer consumers in rich countries who do spend a higher proportion of their income on food and thus who could potentially suffer greater welfare loss from more volatile (higher) prices. It is notable, however, that the high food prices in 2007–2008 were much lower on the political agenda in the rich countries, including Britain, than the high energy and fuel prices.

Despite the inherent risks in agricultural production (Moschini & Hennessy 2001), producers in many richer nations may in principle cope with these risks and the resulting food price volatility through a range of different mechanisms such as forward and futures markets and crop insurance. While these arrangements do little to reduce price volatility, they do allow producers to cope more effectively with this volatility. As such, food price volatility can bring some short-term uncertainty, but in aggregate terms, the welfare impact for producers in richer nations is relatively minor.

Many poorer nations are net importers of food products, either in raw or processed form. For these countries, the proportion of the import bill that goes on food is generally much higher than in richer nations. Grains are the principal commodities of concern, followed by vegetable oils. In Asia, food security concerns relate primarily to the adequacy of rice supplies. In southern and eastern Africa, white maize plays this role. Because many food-importing countries are landlocked, price volatility can be very high—see Dana *et al*. (2006) in relation to maize in Malawi and Zambia. The major use for soya beans is in meat production, so volatility in soya bean prices feeds through into meat prices. This factor is particularly important in China, which is the major world importer of soya beans.

Volatile world food prices can create major import bill uncertainty with concomitant exchange rate uncertainty. Scarce foreign exchange reserves can be exhausted relatively quickly with a sudden spike in food prices as the elasticity of demand for food imports is relatively low. The Food and Agriculture Organization of the United Nations (FAO) (FAO 2008) shows how increasing cereal import costs as a percentage of GDP can lead to a significant widening of the current account deficit in seven economies of more than 3 per cent, while for another seven countries, the anticipated increase is between 2 and 3 per cent (2006/2007–2007/2008).

Many developing country governments act to stabilize the domestic prices of food staples in order to avoid importing volatility from the world market. In most cases, the countries will also be significant producers of the staple. Stabilization will then limit the incentive for domestic farmers to respond to signals from the world market. If a sufficient number of countries act in this way, the resulting reduction in the world supply elasticity will exacerbate volatility. Where countries are net importers, stabilization will require fiscal resources. Food price volatility therefore introduces volatility into government expenditure.

In the poorest nations, where poverty levels are high and where food security becomes a pressing concern, food price volatility can *in extremis* lead to great hardship for consumers and even revolt (the 2008 riots in Indonesia and Haiti, for example), reflecting the fact that food expenditure constitutes a significant proportion of the total income (70–80% of income). Large and sudden increases in prices or indeed, just large increases alone, can ultimately cause hunger, poor nutrition and illness if consumers are unable to buy their staple needs. Equally, as with richer nations, there are potentially inflationary effects in poorer nations too. FAO (2008) shows the relationship between consumer price index (CPI) increases and food price increases for a number of countries, for example, Egypt seeing CPI rise by 15.4 per cent while food prices rose 24.6 per cent (January 2007–January 2008) and Haiti 10.3 and 14.2 per cent, respectively, for the same period.

Clearly, such dramatic impacts on the population are unpalatable for governments who often employ controls on markets or subsidization of prices to mitigate the effects. Controls can take a number of forms, but in periods of very steeply rising prices, some governments have sought to limit food shortages by banning exports of staple products grown in their own country (e.g. rice markets in Vietnam, Cambodia and Egypt). Others try to stem the impact of higher prices by buying at the world market and then selling on to the domestic market at lower (subsidized) prices. The difficulty with this policy is that the expense can cause great stress on government finance as the difference between world and domestic prices gets larger.

## 5. How food price volatility is likely to change in the future

The current concern is that food price volatility may have increased over recent years and may increase further in the future. It follows from the discussion in §2 that an increase in price volatility must arise from one or more of the following four factors:

— an increase in the variance of demand shocks;
— an increase in the variance of supply shocks;
— a decline in the elasticity of demand; and
— a decline in the elasticity of supply.

In §1, we listed a number of factors seen as contributory to food price developments over 2006–2008. In asking whether these factors have had a long-term effect on volatility levels, or whether instead their impact is transient, it is useful to relate the factors to the four categories listed above.

### (a) Increased demand variability

Gilbert (2010*b*) emphasizes the role of demand factors in the determination of food prices, and a number of commentators have pointed to rapid economic growth in China and elsewhere in Asia as the common driver of commodity price changes in energy and metals as well as for foods. If demand growth is becoming more variable as it becomes faster, this will also generate increased food price volatility. At the time of writing, the global *macroeconomic outlook* is highly uncertain and combines continuing fast growth in the emerging economies with a stagnant prospect in the developed economies. If the eventual resolution of current global imbalances involves further crises, these are likely to be reflected in greater food price volatility.

The use of food crops as *biofuel feedstocks* also fits under the demand variability heading. Many commentators have claimed that the demand for food commodities, in particular corn, sugar and vegetable oils, as biofuel feedstocks has increased the correlation between agricultural prices and the oil price—see, in particular, Mitchell (2008). This allows transmission of oil price volatility to agricultural prices, in effect increasing the variance of demand shocks. If one concedes that oil price volatility has increased over time, this could lead to increased food price volatility. There has been no systematic study of the effect of biofuel demand on food price volatility, as distinct from the level of food prices. Scientific studies of the effects of biofuel demand on food price levels fail to find clear evidence of an increased linkage between the oil price and agricultural prices over recent years (Gilbert 2010*a*). This may be because biofuel production in Europe and the United States has to date been driven more by government mandate requirements than by direct profit considerations and has therefore not been sensitive to changes in the oil price. This may change as China becomes a major producer of biofuels.

Index-based investment in commodity futures, discussed in §3 in relation to *speculation*, also relates to the demand variability heading. Index investors purchase long positions in commodity futures, generally via swap transactions, and hold these for extended periods of time. This may be regarded as a form of ‘virtual storage’ in which the investors pay the market to carry inventory on their behalf. The result is to add an additional component to the demand equation and hence also an additional source of demand variability with the implication that financial market shocks can be imported into food markets. Many commercial traders argue that this is precisely what has happened over recent years, with the consequence that price movements have sometimes been divorced from underlying developments in physical supply and demand. Gilbert (2010*a*,*b*) confirms the importance of index-based futures investment in amplifying price movements in 2008 but notes that these effects were smaller in food markets than in energy and metals markets, reflecting the lower involvement of index-based investors in agricultural futures.

### (b) Increased supply variability

Poor Australian wheat harvests in 2006 and 2007 and a poor European 2007 harvest have been mentioned as possible causes of the 2006–2008 food price spike. However, these poor harvests were offset by good harvests elsewhere in the world, notably Argentina, Kazakhstan and Russia, and 2008 harvests were good. Mitchell (2008) discounts poor harvests as a major cause of the spike.

Looking to the future, there must be a concern that *global warming* will increase the variance of agricultural production. Theoretical models, e.g. Schlenker *et al*. (2005) and FAO (2008), suggest damage to existing cropping areas if temperatures rise. It is certainly possible to find clear examples of specific crop–country combinations where this is the case. These mainly relate to production in relatively arid areas—grain production in much of Australia, cattle in areas of Africa bordering the Sahara and food production in South Asia and southern Africa (World Bank 2009). It is widely believed that global warming may result in more extreme weather conditions, and this may result in greater yield variability. We are not aware of scientific discussion of this possibility. In any case, there remains the question of the extent to which increased yield variability in specific crops and countries will generalize to the entire spectrum of food prices.

### (c) Lower demand elasticities

Demand can only respond to price developments if food consumers face prices that are related to world markets. This forces attention on the issue of *food transmission*, i.e. the extent to which prices on world markets are passed through to local prices. Price transmission is generally high in developed countries but, because the food commodity itself often only accounts for a small share of the total value of the product—transportation and marketing dominate—even quite large changes in world prices only have small effects on retail prices. Transmission is more variable in developing countries and is often hindered by high transportation costs that can divorce local prices from those on world markets (Conforti 2004). Over time, greater market integration (‘globalization’) is tending to diminish these barriers. On the other hand, governments often respond to higher food prices by raising subsidies. Irrespective of the wisdom of such policies, they will diminish price responsiveness on the part of consumers. This has been cited as a contributory factor for oil price volatility but has not generally been regarded as important for food crops.

The traditional view of speculation as price stabilizing, discussed in §3, may also be seen as affecting demand elasticities. By buying low and selling high, profitable speculation should reduce price variability. It will do this more effectively as markets become more liquid. There are three qualifications to these arguments. First, the evidence is mixed that speculation is generally profitable (Edwards & Ma 1992, pp. 472–476). Second, not all speculation corresponds to this traditional view—see the discussion of index-based investment in §5*a*. Third, even if speculation does reduce variances at lower frequencies (e.g. month-to-month variability), it also appears to increase higher frequency variances (day-to-day and intraday variability). The overall effects of futures speculation are therefore more mixed than those predicted by the simple traditional account.

### (d) Lower supply elasticities

Grain inventories have fallen over the period since the millennium, and this has been cited as a contributory factor in the 2006–2008 price spike. That argument is difficult to sustain in a simple form since the decline in inventory levels was slow and steady while the price rise, in 2007 and the first half of 2008, was sharp and sudden. What is clearer is that low inventory levels will have reduced the responsiveness of supply to the demand shocks which we argued above are seen as important in generating the price rise. Demand and supply shocks are responsible for the incidence of price changes while the level of inventories determines the amplitude of the resulting price movements.

Grain reserves have fallen to low levels for two reasons. First, commercial users have sought to economize on inventory and have placed reliance on rapid and flexible delivery. Second, governments have come to rely more on trade than food security inventories to meet shortfalls in domestic availability. Both developments have been driven by the awareness that inventories are expensive to maintain. Commercial reliance on suppliers and national reliance on trade provide lower cost solutions to availability problems so long as shocks are idiosyncratic. They will fail when shocks are common. This was brought home to governments in 2008 who found that reliance on trade for food security objectives is likely to fail in exactly those circumstances in which it is required. The result is a move back to inventories both in the commercial supply chain and at the governmental level in relation to food security. Higher grain inventory levels should ensure that future supply and demand shocks are more easily absorbed.

*Underinvestment in agriculture*, cited in World Bank (2007) and particularly acute in the developing world, by contrast, cannot be addressed so rapidly. It takes the form of poor agricultural infrastructure (roads, warehousing, port facilities), undeveloped rural credit, exhaustion of soil nutrients, often as the result of poor farming practice, and lack of research into new seed varieties (Thurow & Kilman 2009). All of these factors limit the ability of developing country farmers to respond to price incentives, and this exacerbates price volatility.

### (e) Exchange rate variability

There is a final factor, exchange rate variability, which does not fit easily into the four categories set out above. Changes in exchange rates reallocate purchasing power and price incentives across countries without changing the overall food supply–demand balance. Dollar depreciation raises prices to US producers and consumers but lowers prices to consumers outside the dollar area. This is because the dollar price of the commodity on world markets will rise as the result of the depreciation, but by less than the extent of the depreciation, implying a fall in say euro and sterling prices (Ridler & Yandle 1972). Exchange rate variability therefore contributes to the variability of prices measured in dollar terms, but would vanish if prices were measured in terms of an appropriately weighted basket of currencies.

The overall scorecard is therefore mixed. Table 3 attempts a highly judgmental summary of the impact of the various factors considered both on the incidence and amplitude of the 2006–2008 price shock and on likely important future price volatility.

**Table 3.** Qualitative importance of different factors.

	2006–2008 impact	likely future impact
the international macroeconomic environment	positive	uncertain
demand for food crops as biofuel feedstocks	positive but small	positive
futures market speculation	positive	positive
climate change	minimal	uncertain
price transmission	minimal	small
inventory levels	positive	small
underinvestment in agriculture	positive but small	positive
exchange rate variability	positive	small

## 6. Rice

Rice, which is the staple food in much of Asia and is also widely imported and consumed in central and west Africa and in the Caribbean, is an exception to many of the general conclusions drawn above in relation to food price volatility.

— Rice is not closely linked in terms of either production or consumption with other major grains—it is produced on different types of land and largely in different countries, and, in the main, is consumed by different groups of consumers.
— Rice production and consumptions shocks are not highly correlated with those in other grains.
— Rice is not currently traded on a liquid futures market—futures markets exist in both Bangkok and Chicago, but they attract relatively little business.

The consequence of these differences is that there is little transmission of price changes from other grains to rice, or *vice versa*. Rice prices therefore tend to follow their own peculiar path. Financial activity on futures markets has little impact. Nevertheless, rice prices did rise strongly in 2007–2008 and remain high in 2009.

The rice story in 2007–2009 is peculiar and in some sense pre-modern (Christiaensen 2009; Timmer 2009). Rice differs from other food commodities in that only a small proportion of world rice enters into international trade (most major consumers are also major producers), and that much rice which is traded is bought or sold at contracted, and not free, market prices. The free market is therefore residual and has the potential to exhibit high volatility. There were no significant production or consumption shocks in the rice market, which was in surplus through the whole of 2007–2008. The initial price rise came in October 2007 when the Indian Government limited rice exports in order to offset the effects of rising wheat prices of the cost of living index. Fears that this might lead to a shortfall led to panic buying by governments of poor rice-importing countries, which drove up prices to unprecedented levels. Prices fell back in July 2008 when the Japanese Government agreed to sell rice from its World Trade Organization (WTO) stockpile. In the end, no rice was sold, but the offer was sufficient to cool the market.

The international rice market is evidently highly problematic as well as politically important—most of the so-called food riots in 2007–2008 involved rice. It is urgent and important that steps are taken to avoid a repeat of this episode (Timmer 2010). In our view, however, it would be an error to see the problems affecting the rice market as generalizing to other grains markets or to wider agricultural markets. Both the sequence of events over 2007–2009 and the volatility statistics in §2 underline that ‘rice is different’. Whether or not rice price volatility increases or declines over the coming years will depend on how well the international community addresses the particular problems of that market, not on any general tendency of volatility in general to increase or decline.

## 7. Review of mechanisms to reduce food price volatility

There have been many attempts to deal with the problems associated with price volatility. These can be reviewed in terms of the time period of interest—the short term and the longer term. Taking the short term first, this refers to an instant and short-term response to increased volatility, often in conjunction with rising price levels. Many developing and middle income countries have sought to deal with significant price volatility either through export controls (as in Southeast Asia in relation to rice) or through price subsidies. The result is that shocks on the world market are not transmitted to domestic consumers. By insulating domestic producers and consumers from what is often seen as ‘imported volatility’, countries reduce demand and supply elasticities in the world market. When a significant number of major producers of the commodity act in this way, prices on the residual world market become highly volatile.

The interesting aspect of these short-term measures is that while domestic markets might experience a degree of greater stability as a result of intervention, the impact on the world market and more open countries is that volatility increases. Such beggar-your-neighbour policies often arise when world markets are in decline or in periods of great instability. This was the situation in the rice market in 2007–2008 and characterized the world sugar market through much of the 1970s and 1980s. In these cases, we need to balance the advantage of reduced volatility in the protected markets against the costs of increased volatility for countries dependent on the residual free market.

Longer term policies and responses are more systematic and expansive in what they try to achieve. At the aggregate level, economies have sought to work collectively to limit fluctuations in world prices of commodities, an approach manifest in the international commodity agreements that dominated the 1960s and 1970s for a range of commodities including sugar, coffee and cocoa. Control in these markets came via a combination of buffer stocks (cocoa) and quota limitation of exports (coffee and sugar) with the aim of maintaining prices within target bands agreed between consumer and producer nations. The historical experience indicates that export controls are politically difficult and cannot easily accommodate the arrival of new producers while buffer stock agreements are costly and vulnerable to speculative attack. Gilbert (1996) argued that the cocoa and sugar agreements achieved little success in their objectives, in the case of cocoa because of lack of adequate financing and in that of sugar because of political problems in relation to the Cuban export quota. The coffee agreement did, however, both raise and stabilize prices and the ending of controls in 1989 resulted in both lower prices and greater volatility.

Coffee market controls lapsed because of a diminished enthusiasm for their enforcement. As the largest coffee-consuming country, the United States saw less interest in supporting the export revenues of its Latin American allies in the post-Cold War period. Brazil, which remains the largest coffee-producing country, had seen its market share eroded by higher cost African producers as the result of export restrictions and, having grown to become the second most important coffee-consuming country, had come to have mixed views on the benefits of high prices (Gilbert 1996). Arguably, if controls had been maintained in 1989, the agreement would have been unable to accommodate the arrival of Vietnam as a major new exporter in the 1990s since this would have required existing exporters to cede export quotas. With the lapse of controls, Vietnamese exports displaced higher cost African production, allowing Brazil to increase its market share.

There have been calls for a return to a more regulated food trade environment as a means of combating some of the effects of world price instability. It is hard, however, to envisage that the current world order would countenance such a move, particularly in a trading environment dominated by multinational trade negotiations designed to create more free trading conditions and which seek to open up markets rather than close them down.

Buffer stock intervention raises different issues. There is a widespread view, discussed in §3, that low levels of grain stocks may have exacerbated food price volatility over 2006–2008. If governments take the view that the private sector is unwilling or unable to hold adequate stocks, they may wish to augment these through public stocks. These could be held either nationally or through an international authority. This policy direction is dangerous. First, public stockholding discourages and crowds out private stockholding (Miranda & Helmberger 1988) as the private sector comes to rely on the availability of subsidized public inventory. The second problem is that any commitment to maintain prices within pre-announced bands, as in the cocoa agreement, makes the stockholding authority vulnerable to speculative attack (Salant 1983). There is a case for public stockholding of food commodities in landlocked developing countries that are largely isolated from world markets and where the private sector is poorly represented. This case is much weaker for developed countries and in relation to the world market where it would be preferable to provide improved incentives for private stockholding. A possible mechanism is for an international agency to purchase grain futures contracts in periods of excess supply so as to induce, and have access to, larger inventories in subsequent years.

Alternative measures for stabilization of price came in *ex post* policies such as the EU's STABEX scheme that focused less on prices *per se* but instead on the impact volatility had on a country's current account balance. Under STABEX, payments were made to those countries that experienced large current account swings owing to increasing import bills or indeed a collapse in export earnings owing to price declines. However, such schemes were often viewed as insensitive to specific country concerns and were quite slow to respond to crises, with the consequence that their impact was probably to amplify rather than dampen the effects of price cycles. The successor FLEX scheme is generally seen as ineffective as while it sought to improve on the STABEX scheme, it still appears to contain some of the constraints and rigidities embodied in its predecessor. As Aiello (2009) suggests, the FLEX scheme has been dogged by a lack of finance to support its operation and also delays in getting funding to those countries that meet eligibility criteria.

In richer nations, agricultural policies have been established often with an explicit target of price volatility reduction, as seen in the original rationale for the EU's Common Agricultural Policy (CAP). While ostensibly more about raising farm incomes, as was also the case in the US policy, the CAP did initially attempt to manage prices for both producers and consumers through elements of supply control. Thus, quotas in sugar and milk, and trade restrictions (import tariffs and export subsidies) sought to balance consumption and production at ‘reasonable’ prices. Much of the policy intervention in recent years (e.g. the reforms under the Macsharry plans of 1992) had been designed to curb the growing subsidization of exports onto world markets as EU production outstripped EU consumption and as the EU came under increasing pressure to negotiate a settlement in the Uruguay round of the GATT talks. Thus, input controls such as set-aside and variable levies were phased out to meet this requirement, although the recent WTO ruling on sugar has led to a reduction in the use of export subsidies in that crop too, which, when coupled with the more generic liberalizing of the EU policy, has led to a more limited ability of the EU to isolate its internal market from the global market.

Instead, greater attention is being paid to market-based measures of price risk management (Morgan 2001). Insurance markets are well developed in most rich nations and offer some cover for crop failure, but not for price risk. Futures and options markets instead provide a means to hedge price risk that is far cheaper than the alternative use of forward contracts and major exchanges in the USA, Britain and increasingly India and China, which offer contracts in a range of major commodities such as grains, soya beans and other soft commodities like sugar, coffee and cocoa. However, direct uptake by producers can be limited (Pannel *et al*. 2007) even when communication is good, awareness of opportunities is high and the advantages would appear strong. At the same time, producers benefit indirectly from the greater pricing that futures-based risk management offers to intermediaries such as grain elevator companies.

In cases where producers do not have such conditions—in poorer nations—use of futures and options markets becomes much more difficult. A World Bank-sponsored project—the International Task Force for Commodity Risk Management (ITF) 2000—sought to explore ways to design intermediation between producer nations and major commodity exchanges so that the benefits of hedging could be opened to all. Dana & Gilbert (2008) review this experience and argue that the major impact is more likely to be seen through the protection of supply chain intermediaries than directly by the producers themselves.

The 2007–2008 food price spike has reawakened interest in food security issues. Governments, whether or not democratic, have found that they cannot afford to leave these issues to the operation of the market. Indeed, the perception on the part of the private sector that governments are unable to commit to staying outside food issues makes it difficult for private traders to ensure adequate supply until government has declared its own hand. In many developing countries, the private sector makes insufficient preparation for food supply problems knowing that government will in the end act, and government does act, justifying the necessity to do so on the basis of the inadequate actions of the private sector. The question is therefore not whether governments should ensure food security, but how they should do so and how they should involve the private sector.

Over the past two decades, western governments and multilateral agencies have emphasized trade over national food reserves. Food reserves were seen as expensive, inflexible and prone to generate corruption. To the extent that supply shocks are uncorrelated across countries, it is less costly to import to meet a domestic shortfall. This advice worked well until 2007 when agricultural prices rose across the board. However, in 2007–2008, exactly when many countries needed to import additional food, they found prices rising against them or, in the extreme case of rice, markets being closed, with the result that supplies were not available at any price. Governments have drawn the conclusion that the advice to rely on trade was incorrect and are now attempting to re-establish food security stocks.

Concerns have been raised about the extent of speculation, and there have been calls for tougher regulation to ensure that supposedly destabilizing speculative activity is controlled. Index-based speculation in commodity futures was highlighted in §5 as a contributory factor in recent food price volatility that may have exacerbated the 2006–2008 food price spike.

— Speculation could be limited by increasing the ‘margin’ required from speculators, thereby increasing their costs. However, this would also limit market liquidity, making it more costly for commercial traders to hedge their risk positions.
— Most exchanges already take steps to limit large positions that may have a price-distorting impact. These measures are commendable but tackle price manipulation more than volatility.
— von Braun & Torero (2009) have proposed a ‘virtual reserve’ system. They suggest that, in the event that speculation drives grains futures prices up to excessive levels, the agency could intervene by selling grains futures—see also von Braun *et al*. (2009). The mere knowledge of this possibility may be sufficient to limit speculative activity. However, lacking the benefits of hindsight, it is very difficult to know whether a particular price level is excessive. There is a clear danger that, instead of discouraging speculation, misjudged interventions may result in the transfer of taxpayers' funds to speculators.
— The most straightforward, and least costly, means of limiting speculation is through encouraging greater transparency in relation to the market situation and, in particular, to stock levels. A number of agencies, in particular the FAO and the International Grains Council, already contribute in this regard, but they are constrained by the information provided to them by national governments. The developed countries of Europe and North America generally provide comprehensive information, but this is not the case for all developing countries, in particular China, which are now major food producers and consumers.

Governments rightly value stability in the prices of basic food commodities. The right balance of policy will vary from commodity to commodity. Many Asian rice-producing countries have long histories of successful stabilization of domestic rice prices using a combination of import and/or export levies and food reserve stockpiles (Dawe 2007; Timmer 2010). However, it seems unlikely that this experience can easily be generalized to the maize and wheat markets where there is greater geographical separation of production and consumption. Furthermore, as we have already noted in discussing the rice price spike, successful domestic price stabilization can often be at the expense of greater volatility in world rice prices, effectively pushing the costs of any shortfall onto many of the world's poorest consumers.

## 8. Conclusions

There is a general tendency for commentators to assert that food price volatility has increased over time—however, the reverse appears to be true. Volatility has jumped over the most recent years, but there have also been periods of high volatility in the past and, except in the important case of grains, the recent episode does not appear exceptional. It is therefore possible to hope that volatility levels will drop back to historical levels over the coming years.

Despite this, there are factors—global warming, oil price volatility transmitted via biofuel demand, index investment in futures markets—that may have led to a permanent increase in volatility in particular in grains prices. We cannot rule this possibility out, but we see little evidence that substantiates these claims, which we therefore regard as (perhaps reasonable) conjecture and not fact. It is unhelpful, but nevertheless correct, to say that we need to wait for several more years before firm conclusions will be possible.

This review has emphasized the exceptionality of rice. Recent rice price volatility has been much greater than historical experience would have suggested as likely. To a considerable extent, perceptions of the recent food price spike were driven by the difficulties experienced in the rice market, and the dramatic price increases that these engendered. Rice was, however, not typical of other markets and the rice experience does not generalize. Low-income rice-importing countries do urgently need to address their food security problems, but the solutions to those problems will not necessarily be relevant to other food commodity markets.

There are three areas in which it would be helpful to have more research.

— Most discussion of climate change in relation to food markets has rightly focused on possible impacts on yields. There has been very little discussion of the possible impact on yield variability.
— We have argued that the biofuel literature has not shown clear links from biofuel production to food prices and from oil price-induced variations in the profitability of biofuels to food price volatility.
— We have highlighted the extensive evidence demonstrating interconnection of financial and food commodity markets as the result of speculative activity. Nevertheless, this contention remains controversial and, until the mechanisms are better understood, the policy debate will remain confused.
